# Supplementary material for: Empirical methods for controlling false positives and estimating confidence in ChIP-Seq peaks
Source: BMC Bioinformatics. 2008 Dec 5;9:523. doi: 10.1186/1471-2105-9-523 (PMC2628906; doi:10.1186/1471-2105-9-523)
Supplement: Additional File 1 — A variety of html documents from the USeq web site detailing the available applications, their best usage, output file type descriptions, command line menus, etc. [file 1471-2105-9-523-S1.zip › USeqUserGuides/JavaDoc.zip!/JavaDoc/util/bio/calc/Alignment.html]

Alignment


|  |  |  |
| --- | --- | --- |
| **Overview** \| **Package** \| **Class** \| **Use** \| **Tree** \| **Deprecated** \| **Index** \| **Help** | |  |
| PREV CLASS   **NEXT CLASS** | **FRAMES**    **NO FRAMES**     **All Classes** |  |
| SUMMARY: NESTED \| FIELD \| CONSTR \| METHOD | DETAIL: FIELD \| CONSTR \| METHOD |  |


---


## util.bio.calc Class Alignment

```
java.lang.Object
  util.bio.calc.Alignment
```

---

``` public class Alignment extends java.lang.Object ```

Methods associated with alignments, pvalues, blast.

---

**Constructor Summary**

`Alignment()`


| **Method Summary** | |
| --- | --- |
| `static int[]` | `calculateEffectiveMandN(double m, double n, double K, double H)`             Calculates effective m and n using BLAST parameters, returning int[m', n'] |
| `static java.lang.String` | `calculateEValue(double lambdaNats, double K, int rawS, double effectiveN, double effectiveM)`             Calculates an E value given a variety of alignment parameters |
| `static double` | `convertBitScoreToRaw(double lambdaNats, double K, double bitS)`             Converts a nat bit score to raw S. |
| `static double` | `convertRawScoreToBit(double lambdaNats, double K, double rawS)`             Converts a raw S score to a bit score given lambda (in nats) and K |
| `static double` | `estimatePValue(double z)`             Calculates the standard normal cumulative distribution given a z-ratio. |
| `static double[]` | `fetchBLASTParams(int match, int misMatch)`             Returns Lambda, K, H or null for a given match and misMatch from BLASTN. |
| `static int` | `ScoreDNAAlignment(java.lang.String seq1, java.lang.String seq2, int match, int misMatch, int gapCreate, int gapExtend)`             Will score a DNA alignment given the two sequences, and scoring parameters. |
| `static double` | `transform10Log10(double num)`             Calculates a -10Log10(number) transformation |

**Methods inherited from class java.lang.Object**

`equals, getClass, hashCode, notify, notifyAll, toString, wait, wait, wait`

**Constructor Detail**

### Alignment

```
public Alignment()
```


**Method Detail**

### estimatePValue

```
public static double estimatePValue(double z)
```

:   Calculates the standard normal cumulative distribution given a z-ratio.
    AKA The area under the curve in the tail.
    Returns 0 or 1 if z>6 or Z<-6, respectively.
    Taken from http://finance.bi.no/~bernt/gcc\_prog/recipes/recipes/node23.html

---


### transform10Log10

```
public static double transform10Log10(double num)
```

:   Calculates a -10Log10(number) transformation

---


### convertRawScoreToBit

```
public static double convertRawScoreToBit(double lambdaNats,
                                          double K,
                                          double rawS)
```

:   Converts a raw S score to a bit score given lambda (in nats) and K

---


### convertBitScoreToRaw

```
public static double convertBitScoreToRaw(double lambdaNats,
                                          double K,
                                          double bitS)
```

:   Converts a nat bit score to raw S.

---


### calculateEValue

```
public static java.lang.String calculateEValue(double lambdaNats,
                                               double K,
                                               int rawS,
                                               double effectiveN,
                                               double effectiveM)
```

:   Calculates an E value given a variety of alignment parameters

---


### calculateEffectiveMandN

```
public static int[] calculateEffectiveMandN(double m,
                                            double n,
                                            double K,
                                            double H)
```

:   Calculates effective m and n using BLAST parameters, returning int[m', n']

---


### fetchBLASTParams

```
public static double[] fetchBLASTParams(int match,
                                        int misMatch)
```

:   Returns Lambda, K, H or null for a given match and misMatch from BLASTN.
    Some combinations of match/misMatch are not computed by BLASTN!

---


### ScoreDNAAlignment

```
public static int ScoreDNAAlignment(java.lang.String seq1,
                                    java.lang.String seq2,
                                    int match,
                                    int misMatch,
                                    int gapCreate,
                                    int gapExtend)
```

:   Will score a DNA alignment given the two sequences, and scoring parameters.


---


|  |  |  |
| --- | --- | --- |
| **Overview** \| **Package** \| **Class** \| **Use** \| **Tree** \| **Deprecated** \| **Index** \| **Help** | |  |
| PREV CLASS   **NEXT CLASS** | **FRAMES**    **NO FRAMES**     **All Classes** |  |
| SUMMARY: NESTED \| FIELD \| CONSTR \| METHOD | DETAIL: FIELD \| CONSTR \| METHOD |  |


---
